# Cerebral Small Vessel Disease Load Predicts Functional Outcome and Stroke Recurrence After Intracerebral Hemorrhage: A Median Follow-Up of 5 Years

**DOI:** 10.3389/fnagi.2021.628271

**Published:** 2021-02-19

**Authors:** Mangmang Xu, Baojin Li, Di Zhong, Yajun Cheng, Qian Wu, Shuting Zhang, Shihong Zhang, Bo Wu, Ming Liu

**Affiliations:** ^1^Department of Neurology, Center of Cerebrovascular Diseases, West China Hospital, Sichuan University, Chengdu, China; ^2^Department of Rehabilitation Medicine, West China Hospital, Sichuan University, Chengdu, China

**Keywords:** cerebral small vessel disease, long-term follow-up, functional outcome, stroke recurrence, cerebral microbleeds

## Abstract

**Background:** Uncertainty exists over the long-term prognostic significance of cerebral small vessel disease (CSVD) in primary intracerebral hemorrhage (ICH).

**Methods:** We performed a longitudinal analysis of CSVD and clinical outcomes in consecutive patients with primary ICH who had MRI. Baseline CSVD load (including white matter hyperintensities [WMH], cerebral microbleeds [CMBs], lacunes, and enlarged perivascular spaces [EPVS]) was evaluated. The cumulative CSVD score was calculated by combining the presence of each CSVD marker (range 0–4). We followed participants for poor functional outcome [modified Rankin scale [mRS] ≥ 4], stroke recurrence, and time-varying survival during a median follow-up of 4.9 [interquartile range [IQR] 3.1–6.0] years. Parsimonious and fuller multivariable logistic regression analysis and Cox-regression analysis were performed to estimate the association of CSVD markers, individually and collectively, with each outcome.

**Results:** A total of 153 patients were included in the analyses. CMBs ≥ 10 [adjusted OR [adOR] 3.252, 95% CI 1.181–8.956, *p* = 0.023] and periventricular WMH (PWMH) (adOR 2.053, 95% CI 1.220–3.456, *p* = 0.007) were significantly associated with poor functional outcome. PWMH (adOR 2.908, 95% CI 1.230–6.878, *p* = 0.015) and lobar CMB severity (adOR 1.811, 95% CI 1.039–3.157, *p* = 0.036) were associated with stroke recurrence. The cumulative CSVD score was associated with poor functional outcome (adOR 1.460, 95% CI 1.017–2.096) and stroke recurrence (adOR 2.258, 95% CI 1.080–4.723). Death occurred in 36.1% (13/36) of patients with CMBs ≥ 10 compared with 18.8% (22/117) in those with CMB < 10 (adjusted HR 2.669, 95% CI 1.248–5.707, *p* = 0.011). In addition, the cumulative CSVD score ≥ 2 was associated with a decreased survival rate (adjusted HR 3.140, 95% CI 1.066–9.250, *p* = 0.038).

**Conclusions:** Severe PWMH, CMB, or cumulative CSVD burden exert important influences on the long-term outcome of ICH.

## Introduction

Spontaneous primary intracerebral hemorrhage (ICH) is mainly caused by cerebral small vessel disease (CSVD), including hypertensive arteriopathy (HA) and cerebral amyloid angiopathy (CAA) (Charidimou et al., 2016; Hankey, 2017), and has both short- and long-term consequences of high case fatality, disability, and risk of recurrent serious vascular events (Poon et al., 2014). Both HA and CAA are associated with magnetic resonance imaging (MRI) markers of CSVD, including lacunes, white matter hyperintensities (WMH), cerebral microbleeds (CMBs), and enlarged perivascular spaces (EPVS) (Charidimou et al., 2016). These MRI markers could reflect the presence and severity of CSVD-related damage (Wardlaw et al., 2013) and rarely occur in isolation. Thus, several semiquantitative scales have been developed to assess the cumulative CSVD burden (Staals et al., 2014; Xu et al., 2018).

Numerous studies have investigated the predictors of mortality and functional outcome following ICH (Gregório et al., 2018), whereas few have evaluated the role of CSVD in clinical outcomes after ICH (Sykora et al., 2017; Lioutas et al., 2018). At day 90, the cumulative CSVD burden and the presence of CMB (Lioutas et al., 2018), as well as severe WMH and increasing lacune number (Sykora et al., 2017), are important determinants of poor outcomes. Another study has shown that severe leukoaraiosis on CT scan is significantly associated with a poor functional outcome at discharge and with less recovery in mRS from discharge to day 90 (Uniken Venema et al., 2019).

However, to our knowledge, the effect of the CSVD burden on MRI on the long-term prognosis after ICH (for instance, beyond 1 year) has not been studied. Insights into the longitudinal association between baseline CSVD burden, individually and collectively, and long-term prognosis could possibly lead to a better appraisal of ICH and aid in the development of new therapeutic and preventative strategies to relieve the disease burden.

To address this gap, we aimed to investigate the association between individual CSVD markers and cumulative CSVD burden and (1) poor functional outcome, (2) stroke recurrence, and (3) time-varying survival.

## Methods

### Study Design

We analyzed consecutive patients with primary ICH with MRI susceptibility-weighted imaging (MRI-SWI), admitted to either the Department of Neurosurgery or the Department of Neurology, West China Hospital, Sichuan University from January 2012 to October 2017 in a prospectively collected database. Our study was approved by the Ethics Committee on Biomedical Research, West China Hospital of Sichuan University. Informed consents were obtained from patients or their family members.

### Patient Selection

A total of 203 consecutive patients with primary ICH with MRI-SWI examination in our ongoing prospective cohort study were screened. For our present analysis, patients were eligible if they (1) meet the diagnosis of primary ICH and (2) had MRI with adequate sequences at baseline for the assessments of lacunes, CMBs, WMH, and EPVS. Patients were excluded if (1) the hemorrhage was considered to be primary intraventricular hemorrhage (IVH), (2) the time of onset was beyond 3 months of symptom onset, (3) they had hemorrhagic transformation after an ischemic stroke, (4) their images were of poor quality, or (5) the time of onset was unclear. Angiography was carried out when clinical characteristics were suggestive of a vascular malformation.

### ICH Subtype Classification

The ICH subtypes (HA-ICH, CAA-ICH, mixed-location ICH, and undetermined-ICH) were coded according to the previous studies (Linn et al., 2010; Pasi et al., 2018). We defined individuals as having HA-ICH when patients had deep ICH, with or without deep CMBs, but no lobar ICH or lobar CMBs. Those with strictly lobar ICH (cerebellar hemorrhage allowed) without deep ICH or CMBs were coded as having CAA-ICH when they aged ≥55 years and as undetermined-ICH when they were younger than 55 years. ICH or CMBs in both deep and lobar regions were coded as mixed-location ICH.

### Data Collection

We collected age, sex, vascular risk factors (including hypertension, diabetes mellitus, hyperlipidemia, cardiac disease, smoking, and alcohol consumption) (Valenti et al., 2016), a previous history of stroke, stroke severity [evaluated using Glasgow Coma Scale [GCS] score] on admission, serum factors (including blood glucose, albumin, cholesterol, high-density lipoprotein, low-density lipoprotein, and creatinine), and treatment (surgical hematoma evacuation vs. conservative treatment) for each enrolled participant at baseline. We also collected 10 prespecified complications recorded by trained neurologists, which included pneumonia, urinary tract infection, gastrointestinal bleeding, seizure, septic shock, electrolyte disturbances, deep vein thrombosis, other infections, depression, and anxiety. A list of complications and their definitions are provided in Supplementary Table 1. The definition of each complication was the same as that in the previous studies (Langhorne et al., 2000; Indredavik et al., 2008; Dellinger et al., 2013; Li et al., 2019). We did not include chronic medical complications such as chronic chest infection, since they were beyond the scope of this study.

### Imaging Acquisition and Analysis

#### Computed Tomography

Neurologists who were blinded to the clinical information of patients assessed the ICH location, the ICH volume, the presence of IVH, and midline shift. The ICH location was classified into lobar (hemorrhage originating at the cortex and subcortical junction), deep [hemorrhage located in the basal ganglia (BG), thalamus, or brain stem], or cerebellar ICH. We grouped cerebellar and brain stem ICH into infratentorial ICH. The ICH volume at baseline was assessed using the ABC/2 method (Kothari et al., 1996).

#### Magnetic Resonance Imaging

The median time from the onset to MRI examination was 6.4 [interquartile range [IQR] 3.9–15.8] days in our present study. All enrolled patients underwent MRI scanning, including T1- and T2-weighted, fluid-attenuated inversion recovery (FLAIR), and SWI sequences, with a magnetic field strength of 3 T. The details of MRI parameters of the four sequences were described in our previous studies (Xu et al., 2018, 2019).

A lacune of presumed vascular origin was defined as a small lesion (3–20 mm in diameter) and CSF-like intensity with hyperintensities on T2-weighted and hypointensities on T1-weighted images, with a perilesional halo on FLAIR images sometimes (Klarenbeek et al., 2013; Staals et al., 2014). WMH was assessed using the Fazekas scale, which was a simple scale rating separately for the deep and periventricular region on a 4-point scale ranging from 0 to 3 (Fazekas et al., 1987). We summed the deep WMH (DWMH) score and the periventricular WMH (PWMH) score to form a total WMH score (scored 0–6). The presence of WMH was defined as PWMH Fazekas 3 or/and DWMH Fazekas 2–3(Staals et al., 2014). A CMB lesion was defined as a small area of hypointense signal void on SWI sequences, round or ovoid, blooming, and at least partly surrounded by the brain parenchyma, in line with the current consensus criteria (Greenberg et al., 2009). We recorded the CMB number and their topographic distributions. The lobar CMB severity was defined as follows: 0 = absence of lobar CMB, 1 = 1–4 lobar CMBs, 2 = 5–9 lobar CMBs, 3 = 10–19 lobar CMBs, and 4 = equal to or more than 20 lobar CMBs. Deep CMB severity was defined as follows: 0 = absence of deep CMB, 1 = 1–4 deep CMBs, 2 = 5–9 deep CMBs, 3 = 10–19 deep CMBs, and 4 = equal to or more than 20 deep CMBs. CMB mimics, such as calcium and iron deposits, were excluded using CT scans (Greenberg et al., 2009). EPVS were defined as fluid-filled spaces, linear, round, or ovoid, of <3 mm in diameter and were evaluated on the axial T2-weighted images. The side of the slice with the highest EPVS number in BG and centrum semiovale (CSO) was recorded after reviewing all relevant slices for the two anatomical areas (Wardlaw et al., 2013).

The cumulative CSVD score was calculated using an ordinal 5-point scale ranging from 0 to 4 by combining the four individual CSVD markers, with one point allocated to each of the following: the presence of any lacune, the presence of any CMB, the presence of WMH, and BG EPVS > 10 according to the previous studies (Staals et al., 2014; Song et al., 2017). We defined severe cumulative CSVD burden when the CSVD score was equal to or greater than the median value.

### Outcomes

The primary outcome in this study was a poor functional outcome [the Modified Rankin Scale [mRS] ≥ 4, which means severe functional dependence or death] at the end of the follow-up. Prespecified second outcomes included stroke recurrence and time-varying survival. We followed patients with telephone interviews at 3-months, 6-months, 1-year, and ≥3-years post-ICH. All patients were followed up from enrollment, until the occurrence of death, the last clinical documentation, or the end of the last follow-up (April 2020). A stroke recurrence (including either ischemic or hemorrhagic stroke) was confirmed by asking patients or their family members. We confirmed a stroke recurrence by asking if the patient went to medical institutions (both inpatient and outpatient settings) because of any episodes of new weakness or numbness in legs or/and arms, or new problems with speech or vision, with a new diagnosis of stroke (ischemic stroke or ICH) by specialists.

### Statistical Analysis

Interrater agreement of the CSVD markers was good or excellent, as previously described (Xu et al., 2019). We used the Pearson's chi-squared test or the Fisher's exact test for categorical variables, and the Student *t*-test or the Mann–Whitney *U* test for continuous variables, as appropriate. First, the association between risk factors and each outcome was estimated using the univariate analysis. Then, factors with *p* < 0.2 were forced into multivariable analyses as covariates to determine the association of each individual CSVD marker and the cumulative CSVD score with clinical outcomes. We used multivariable logistic regression analysis to determine the association between CSVD and poor functional outcome and stroke recurrence and multivariable Cox proportional hazard regression analysis to assess the relationship of CSVD with time-varying survival. Potential risk factors, which were entered into the multivariable analysis of the association between CSVD and poor functional outcome, included age (continuous variable), sex, hypertension, cardiac disease, the GCS score (continuous variable), serum albumin (continuous variable), and any complication. In addition, we adjusted for IVH and infratentorial ICH further since the two were the commonly used factors of ICH prognosis in a recent meta-analysis (Gregório et al., 2018). In the multivariable analysis of the association of CSVD with stroke recurrence, we only included patients who were first-ever ICH and had data of stroke recurrence, adjusting for age, sex, and ICH subtypes. We used the receiver operating characteristic (ROC) curve and the area under the ROC curve (AUC) value for each MRI maker and the cumulative CSVD score to characterize the discrimination for poor functional outcome and stroke recurrence. DeLong et al. test was used to evaluate the differences between two AUC values for significance (DeLong et al., 1988). IBM SPSS Statistics (version 21) and MedCalc (version 19.1) were used for statistical analyses. *p* < 0.05 was considered to be significant.

## Results

**Figure 1:**
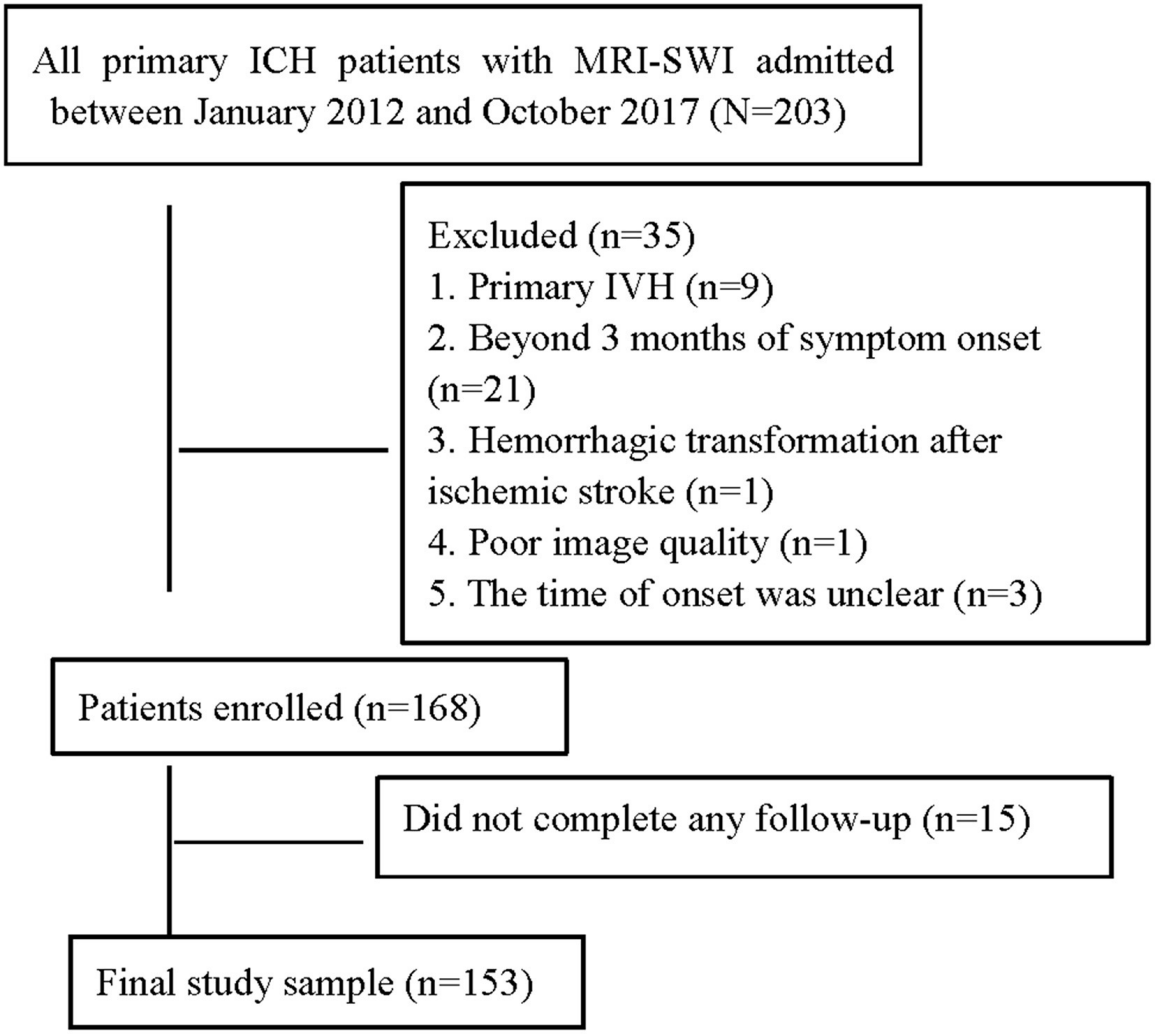
Flow chart of patient selection.

A total of 203 patients with primary ICH and MRI-SWI examination were screened, and 35 out of 203 (17.2%) patients were excluded for various reasons (see Figure 1). Thus, 168 (82.8%; 61.0 ± 12.2 years, age range = 36–88 years) patients were enrolled in our present study. During a median follow-up of 4.9 (IQR 3.1–6.0) years, 15 participants did not complete any follow-up, leaving 153 patients who were included in the analyses (Figure 1). Of these, 111 (72.5%) were men; the most common ICH subtype was mixed-location ICH (43.1%), followed by HA-ICH (39.9%), and CAA-ICH (15.0%). Three patients younger than 55 years with strictly lobar (or cerebellar) ICH and without deep CMB were classified into the undetermined subgroup.

Comparing with those who were lost to follow-up, completers had a lower GCS score (*p* = 0.029) and a lower rate of diabetes mellitus (*p* = 0.025). Other clinical characteristics, serum factors, individual CSVD marker, and the cumulative CSVD score were comparable between the two groups (see Supplementary Table 2).

### Primary Outcome

#### Association Between CSVD and Poor Functional Outcome

At the end of follow-up, 30.1% (46/153) developed poor functional outcome, older (*p* < 0.001), having lower GCS score (*p* = 0.004), and lower serum albumin levels (*p* = 0.001), as well as higher prevalence of any complication (*p* = 0.001) than those who did not develop poor functional outcome. The overall characteristics of the 153 patients in the poor functional outcome analysis were shown in Supplementary Table 3.

The univariate analysis showed that patients with poor functional outcome had a greater proportion of any lacune, more severe PWMH, DWMH, and total WMH score, as well as a greater proportion of CMB ≥ 10 and BG EPVS > 20, and expectedly higher cumulative CSVD score (all *p* < 0.05) compared with those without a poor functional outcome (see Table 1).

**Table 1:** Comparison between patients with and without poor functional outcome and between those with and without stroke recurrence after ICH.

**Variables**	**Poor functional outcome (*****n*** **=** **153)**			**Stroke recurrence (*****n*** **=** **131)**		
	**No** **(*n* = 107)**	**Yes** **(*n* = 46)**	***p***	**No** **(*n* = 117)**	**Yes** **(*n* = 14)**	***p***
Age, y; mean ± SD	58.0 ± 11.5	69.3 ± 10.3	<0.001	61.0 ± 12.6	63.2 ± 11.0	0.529
Male, *n* (%)	73 (68.2)	38 (82.6)	0.068	88 (75.2)	10 (71.4)	0.750
**ICH SUBTYPES**, ***n*** **(%)**						
HA-ICH	46 (43.0)	15 (32.6)	0.387	53 (45.3)	2 (14.3)	0.006
CAA-ICH	16 (15.0)	7 (15.2)		17 (14.5)	1 (7.1)	
Mixed-location ICH	42 (39.3)	24 (52.2)		46 (39.3)	9 (64.3)	
Undetermined	3 (2.8)	0		1 (0.9)	2 (14.3)	
**ICH CHARACTERISTICS**						
Infratentorial ICH, *n* (%)	16 (15.0)	8 (17.4)	0.704	17 (14.5)	4 (28.6)	0.239
GCS score^*^, mean ± SD	13.7 ± 2.3	12.2 ± 3.0	0.004	13.3 ± 2.6	12.8 ± 3.1	0.479
Hematoma volume^†^, median (IQR)	9.4 (3.0–19.7)	10.0 (3.7–24.3)	0.977	10.0 (4.2–18.8)	10.1 (3.6–24.2)	0.960
IVH, *n* (%)	24 (22.4)	12 (26.1)	0.625	27 (23.1)	6 (42.9)	0.116
Midline shift, *n* (%)	27 (25.2)	15 (32.6)	0.349	33 (28.2)	4 (28.6)	1.000
Surgical hematoma evacuation, *n* (%)	12 (11.2)	2 (4.3)	0.231	11 (9.4)	1 (7.1)	1.000
**VASCULAR RISK FACTORS AT BASELINE**						
Systolic BP, mean ± SD	156.8 ± 28.0	159.3 ± 29.5	0.622	159.4 ± 29.4	159.9 ± 24.0	0.949
Diastolic BP, mean ± SD	93.8 ± 15.9	90.3 ± 15.4	0.209	93.8 ± 16.7	89.6 ± 12.2	0.372
Hypertension, *n* (%)	89 (83.2)	34 (73.9)	0.186	95 (81.2)	13 (92.9)	0.462
Diabetes mellitus, *n* (%)	10 (9.3)	6 (13.0)	0.567	13 (11.1)	1 (7.1)	1.000
Hyperlipidemia, *n* (%)	7 (6.5)	1 (2.2)	0.436	6 (5.1)	0	1.000
Prior stroke, *n* (%)	12 (11.2)	3 (6.5)	0.555	NA	NA	
Smoking, *n* (%)	30 (28.0)	16 (34.8)	0.404	36 (30.8)	5 (35.7)	0.763
Alcohol consumption, *n* (%)	20 (18.7)	8 (17.4)	0.849	22 (18.8)	2 (14.3)	1.000
Cardiac disease, *n* (%)	4 (3.7)	6 (13.0)	0.067	7 (6.0)	1 (7.1)	1.000
**LABORATORY TEST, MEAN** ± **SD**						
Blood glucose^‡^	6.9 ± 2.1	7.0 ± 2.4	0.867	7.0 ± 2.3	6.9 ± 1.7	0.783
Albumin^‡^	42.5 ± 4.0	39.9 ± 4.8	0.001	41.9 ± 4.4	42.6 ± 3.1	0.598
Cholesterol^‡^	4.4 ± 0.9	4.4 ± 0.9	0.788	4.5 ± 0.9	4.5 ± 1.1	0.968
HDL^‡^	1.4 ± 0.5	1.5 ± 0.5	0.473	1.5 ± 0.5	1.5 ± 0.4	0.750
LDL^‡^	2.6 ± 0.8	2.5 ± 0.6	0.563	2.6 ± 0.7	2.6 ± 1.0	0.938
Creatinine^‡^	82.9 ± 44.9	83.1 ± 24.0	0.981	80.8 ± 33.4	85.2 ± 24.8	0.630
**COMPLICATION AT BASELINE**, ***n*****(%)**						
Any complication	15 (14.0)	17 (37.0)	0.001	22 (18.8)	2 (14.3)	1.000
**CSVD Severity At Baseline**, ***n*****(%) Or Median (IQR) When Appropriate**						
Lacune ≥ 1	42 (39.3)	27 (58.7)	0.027	47 (40.2)	10 (71.4)	0.026
Lacune ≥ 2	27 (25.2)	10 (21.7)	0.643	25 (21.4)	6 (42.9)	0.096
Lacune number	0 (0–2)	1 (0–1)	0.122	0 (0–1)	1 (0–2.5)	0.023
The presence of WMH	42 (39.3)	31 (67.4)	0.001	49 (41.9)	11 (78.6)	0.009
PWMH score	1 (1–2)	3 (1–3)	<0.001	1 (1–2)	3 (1.8–3)	0.002
DWMH score	1 (1–2)	2 (1–3)	0.003	1 (1–2)	2 (0.8–3)	0.048
Total WMH score	2 (2–4)	4 (2.8–6)	<0.001	2 (2–4)	5 (3.3–6)	0.011
The presence of CMB	75 (70.1)	38 (82.6)	0.106	83 (70.9)	12 (85.7)	0.348
CMBs ≥ 5	36 (33.6)	23 (50.0)	0.057	42 (35.9)	6 (42.9)	0.610
CMBs ≥ 10	20 (18.7)	16 (34.8)	0.031	24 (20.5)	6 (42.9)	0.088
Lober CMB number	0 (0–2)	1 (0–3.3)	0.156	0 (0–2)	2 (0–18.8)	0.012
BG EPVS > 10	51 (47.7)	26 (56.5)	0.315	57 (48.7)	9 (64.3)	0.271
BG EPVS > 20	19 (17.8)	17 (37.0)	0.010	24 (20.5)	5 (35.7)	0.303
CSO EPVS > 20	34 (31.8)	14 (30.4)	0.870	35 (29.9)	3 (21.4)	0.756
Cumulative CSVD score	2 (1–3)	3 (2–4)	0.002	2 (1–3)	3 (2–4)	0.006

ICH, intracerebral hemorrhage; HA, hypertensive arteriopathy; CAA, cerebral amyloid angiopathy; GCS, Glasgow Coma Scale; BP, blood pressure; SD, standard deviation; IQR, interquartile range; IVH, intraventricular hemorrhage; HDL, high-density lipoprotein; LDL, low-density lipoprotein; CSVD, cerebral small vessel disease; WMH, white matter hyperintensities; PWMH, periventricular WMH; DWMH, deep WMH; EPVS, enlarged perivascular spaces; BG, basal ganglia; CSO, centrum semiovale; CMB, cerebral microbleed.

* 152 patients had data of the GCS score in the analysis of poor functional outcome and 130 in the analysis of stroke recurrence.

† 119 patients had data of hematoma volume in the analysis of poor functional outcome and 106 in the analysis of stroke recurrence.

‡ 150 patients had data of cholesterol, HDL, and LDL in the analysis of poor functional outcome and 129 patients had those data in the analysis of stroke recurrence.

§ *152 patients had data of blood glucose, albumin, and creatinine in the analysis of poor functional outcome*.

After adjusting for the prespecified confounders higher PWMH score [adjusted OR[adOR] 2.085, 95% CI 1.255–3.462, *p* = 0.005], higher total WMH score (adOR 1.395, 95% CI 1.071–1.819, *p* = 0.014), CMB ≥ 5 (adOR 2.885, 95% CI 1.162–7.163, *p* = 0.022), CMB ≥ 10 (adOR 3.249, 95% CI 1.201–8.794, *p* = 0.020), and higher cumulative CSVD score (adOR 1.460, 95% CI 1.017–2.096, *p* = 0.040) were all significantly associated with poor functional outcome. The unfavorable prognostic effect of high CMB burden, PWMH severity on poor functional outcome was retained after adjusting for IVH and infratentorial ICH additionally (adOR 2.053, 95% CI 1.220–3.456, *p* = 0.007 for PWMH score; adOR 3.252, 95% CI 1.181–8.956, *p* = 0.023 for CMBs ≥ 10) (see Table 2).

**Table 2:** Association of CSVD with poor functional outcome in the multivariable logistic regression analysis.

	**Mode l**				**Model 2**			
	***p***	**OR**	**95% C**		***p***	**OR**	**95% CI**	
			**Lower**	**Upper**			**Lower**	**Upper**
**INDIVIDUAL CSVD Marker**								
Lacune ≥ 1	0.078	2.164	0.918	5.100	0.081	2.159	0.908	5.132
The presence of WMH	0.065	2.382	0.947	5.992	0.109	2.157	0.843	5.516
PWMH score	0.005	2.085	1.255	3.462	0.007	2.053	1.220	3.456
DWMH score	0.092	1.505	0.936	2.420	0.134	1.446	0.892	2.345
Total WMH score	0.014	1.395	1.071	1.819	0.022	1.373	1.047	1.801
CMB ≥ 1	0.056	2.884	0.972	8.554	0.074	2.722	0.908	8.162
CMB ≥ 5	0.022	2.885	1.162	7.163	0.020	2.984	1.187	7.500
CMB ≥ 10	0.020	3.249	1.201	8.794	0.023	3.252	1.181	8.956
BG EPVS > 20	0.707	1.208	0.451	3.239	0.868	1.089	0.399	2.968
**COMBINED CSVD BURDEN**								
Cumulative CSVD score	0.040	1.460	1.017	2.096	0.073	1.406	0.969	2.039
CMB ≥ 10+ PWMH	0.001	2.124	1.352	3.337	0.002	2.109	1.327	3.352

Model 1: Adjusted for age per year, sex, hypertension, cardiac disease, GCS per unit, serum albumin per unit, and any complication for each individual CSVD marker and each CSVD score.

Model 2: Adjusted for model 1 + IVH and infratentorial ICH.

Assignment:

Cumulative CSVD score: one point allocated to each of the following markers: the presence of lacune, CMB, and the presence of WMH and BG EPVS > 10.

*CMB ≥ 10 + PWMH: CMB ≥ 10 (0 = absence, 1 = presence), PWMH score (Fazekas scale ranging from 0 to 3)*.

Then, we combined CMB ≥ 10 (0 = absence, 1 = presence) and PWMH score (Fazekas scale ranging 0–3) into a single score. With every point increase in this score, the risk of poor functional outcome increased by 2.1 times (adOR 95% CI 1.327–3.352, *p* = 0.002) (Table 2). The predictive value of CMB ≥ 10+ PWMH for poor functional outcome performed better than the individual marker of CMB ≥ 10 (CMB ≥ 10+ PWMH vs. CMB ≥ 10, AUC 0.704 vs. 0.580, *p* = 0.003) or CMB ≥ 5 (CMB ≥ 10+ PWMH vs. CMB ≥ 5, AUC 0.704 vs. 0.582, *p* = 0.007) (Supplementary Table 4).

### Secondary Outcomes

#### Association Between Cerebral Small Vessel Disease and Stroke Recurrence

Of the 153 patients with mRS data, 15 had a previous history of stroke, and seven had missing data on recurrence, leaving 131 first-ever ICH patients who were included in the stroke recurrence analysis. During a median follow-up of 4.9 years, 14 patients experienced recurrent stroke (16 recurrent events overall; seven ICHs, six ischemic strokes, and three unclear stroke subtypes). The recurrence rate in HA-ICH, CAA-ICH, and mixed-location ICH was 3.5% (2/57), 5.6% (1/18), and 17.0% (9/53), respectively. The risk of stroke recurrence varied by ICH subtypes (*p* = 0.006, Fisher's exact test).

Compared to patients without stroke recurrence, recurrent patients were more likely to present with lacunes, WMH, lobar CMB, and a higher cumulative CSVD score in the univariate analysis (all *p* <0.05) (Table 1). After adjusting for age, sex, and ICH subtypes, the unfavorable prognostic effect of PWMH (adOR 2.908, 95% CI 1.230–6.878; *p* = 0.015), total WMH (adOR 1.602, 95% CI 1.046–2.455; *p* = 0.030), lobar CMB severity (adOR 1.811, 95% CI 1.039–3.157; *p* = 0.036), and the cumulative CSVD score (adOR 2.258, 95% CI 1.080–4.723; *p* = 0.030) on stroke recurrence was retained.

Similarly, we combined PWMH (Fazekas scale ranging from 0 to 3) and lobar CMB severity into a single score. The association between this score and stroke recurrence was substantially significant (adOR 1.847, 95% CI 1.194–2.858, *p* = 0.006) (Table 3). The predictive value of the combined CSVD burden (cumulative CSVD score or PWMH + lobar CMB severity) for stroke recurrence was not significantly different from that of an individual marker (Supplementary Table 4).

**Table 3:** Association of CSVD with stroke recurrence in multivariable logistic-regression.

	**Age** **+** **sex adjusted**				**Age** **+** **sex** **+** **ICH subtypes adjusted**			
	***p***	**OR**	**95% CI**		***p***	**OR**	**95% CI**	
			**Lower**	**Upper**			**Lower**	**Upper**
**INDIVIDUAL CSVD Marker**								
The presence of lacune	**0.039**	3.663	1.068	12.558	0.116	3.168	0.753	13.323
Lacune number	0.068	1.266	0.982	1.632	0.183	1.213	0.913	1.611
The presence of WMH	0.017	5.968	1.381	25.793	0.057	5.474	0.948	31.603
PWMH score	0.003	3.127	1.457	6.711	0.015	2.908	1.230	6.878
DWMH score	0.054	1.889	0.988	3.611	0.151	1.714	0.822	3.573
Total WMH score	0.008	1.672	1.146	2.439	0.030	1.602	1.046	2.455
The presence of CMB	0.276	2.386	0.500	11.396	0.416	2.615	0.258	26.506
Lobar CMB severity	0.009	1.760	1.153	2.687	0.036	1.811	1.039	3.157
Deep CMB severity	0.111	1.424	0.922	2.199	0.200	1.409	0.834	2.380
**COMBINED CSVD Burden**								
Cumulative CSVD score	0.009	2.151	1.207	3.834	0.030	2.258	1.080	4.723
PWMH + lobar CMB severity	0.001	1.787	1.263	2.526	0.006	1.847	1.194	2.858

Assignment of deep CMB severity: 0 = absence of deep CMB, 1 = 1–4 deep CMBs, 2 = 5–9 deep CMBs, 3 = 10–19 deep CMBs, 4 = equal to or more than 20 deep CMBs.

*Assignment of lobar CMB severity: 0 = absence of lobar CMB, 1 = 1–4 lobar CMBs, 2 = 5–9 lobar CMBs, 3 = 10–19 lobar CMBs, 4 = equal to or more than 20 lobar CMBs*.

#### CSVD Burden and Time-Varying Survival

**Figure 2:**
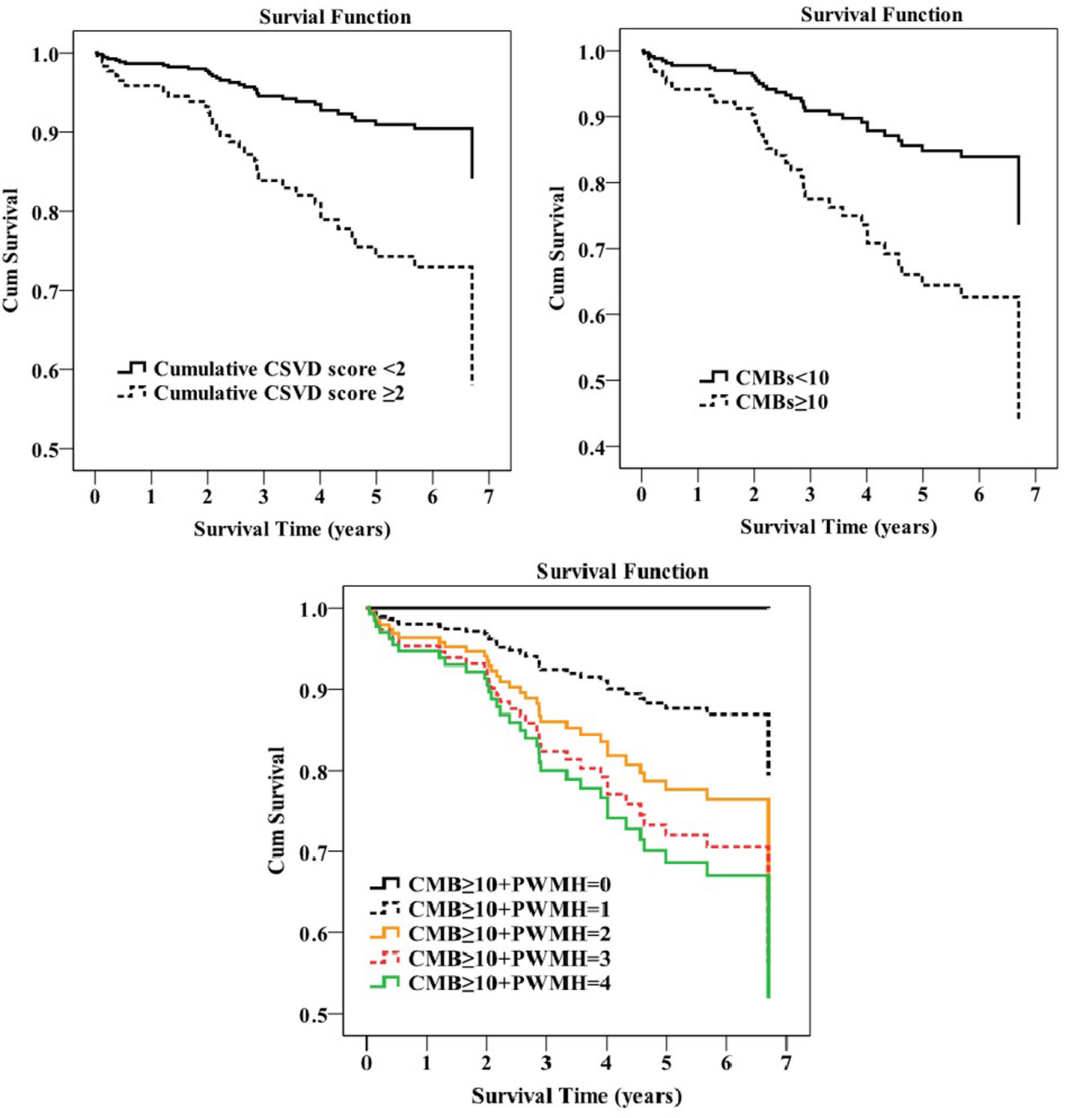
Survival plot of how survival was influenced by the cerebral small vessel disease (CSVD) burden in the Cox regression analysis.

The median value of the cumulative CSVD score was 2 (1–3); so, the prespecified high cumulative CSVD burden was defined as the cumulative CSVD score ≥ 2. The Cox proportional hazards regression analysis showed that only CMBs ≥ 10 was significantly related to mortality (adjusted HR 2.669, 95% CI 1.248–5.707; *p* = 0.011) after adjusting for age, sex, hypertension, cardiac disease, GCS, albumin, and any complication. Additionally, a higher combined CSVD burden was associated with a decreased survival rate (adjusted HR 3.140, 95% CI 1.066–9.250, *p* = 0.038 for cumulative CSVD score ≥ 2; adjusted HR 1.457, 95% CI 1.049–2.024, *p* = 0.025 for CMB ≥ 10 + PWMH) (Figure 2). We found no statistically significant differences in survival, stratified by lacune, PWMH, DWMH, or EPVS.

## Discussion

The key findings of this study are that severe PWMH and CMB were independent predictors of poor functional outcome. There was a substantial risk of stroke recurrence in patients with greater PWMH or lobar CMB burden in patients with ICH. As expected, cumulative CSVD burden altered the occurrence risk of each of the three prespecified outcomes in long-term follow-up, mainly because of the comprehensive effect of WMH and CMB. The predictive value of the combined score (CMB ≥ 10 + PWMH) for poor functional outcome performed better than that of the individual marker of CMB ≥ 10 or CMB ≥ 5.

In our present study, severe PWMH were significantly associated with poor functional outcome and stroke recurrence, independent of age and other potential confounders. Patients with a high PWMH score were older (*p* < 0.001, anova) and more likely to have hypertension (*p* < 0.05, the chi-squared test) than those with a low PWMH score. Severe PWMH was significantly associated with the presence of mixed-location ICH (*p* < 0.001, the chi-squared test) in this study. As shown in the previous literature, mixed ICHs were more likely to have pronounced classic vascular risk factor burden (hypertension, diabetes mellitus, and creatinine) and high WMH volume compared to HA-ICH or CAA-ICH (*p* < 0.001) (Pasi et al., 2018), suggesting that the burden of classic vascular risk factor could be a critical player in the development of a severe arteriopathy in the brain. The mechanism underlying why PWMH, but not DWMH, was associated with a poor outcome is not clear. Perhaps, the vascular architecture in periventricular white matter is more susceptible to damage than that in other areas (Pantoni and Garcia, 1997). Anatomically, there is a high density of long association fibers connecting the cortex with distant brain territories and subcortical nuclei, while the subcortical region has a high density of short-looped U-fibers, which connect adjacent regions (Filley, 1998; De Groot et al., 2002). Lesions affecting long association fibers may have more influence on function than lesions affecting connectivity between neighboring brain areas because of possibly reserve mechanisms of the latter (De Groot et al., 2002).

The strong association of CMB with the long-term poor functional outcome is a novel observation. Lioutas et al. (2018) reported a significant detrimental effect of CMB on 90-days functional independence (mRS ≤ 2) (Lioutas et al., 2018). Our study extended the follow-up to a median of 5 years, verifying the detrimental effect of CMB on long-term prognosis, and further found that PWMH, not DWMH, was closely associated with a poor outcome and stroke recurrence.

The association between the lobar CMB severity and the stroke recurrence is expected. Histopathologically, CMB corresponds with the focal deposition of hemosiderin surrounding small vessels (Greenberg et al., 2009). Those lobar CMBs, which might be due to CAA (Greenberg et al., 2009), are correlated to a greater beta-amyloid burden than deep CMBs (Tsai et al., 2017). A previous study showed that the lobar CMB number was associated with the recurrence of lobar ICH during a median follow-up of 34.3 months (Biffi et al., 2010). A pooled analysis of 10 studies also found that multiple baseline CMBs were associated with the recurrence during follow-up (Charidimou et al., 2017).

### Strength and Limitations

First, we used 3-T SWI to detect CMB. Second, although recurrence was more common in the lobar ICH than in the deep ICH as per the previous systematic reviews (Bailey et al., 2001; Poon et al., 2014), probably because of the type and severity of the underlying microangiopathies (Pantoni, 2010), few studies have systematically addressed the phenotype of the underlying small vessel disease type (Bailey et al., 2001; Poon et al., 2014). In our present study, we used MRI which is the most useful and non-invasive method to phenotype microangiopathies (Charidimou et al., 2017) to directly control the association between CSVD burden and stroke recurrence. Third, our follow-up was relatively long. As reported in a recent study, management in the stroke unit or neurointensive care unit might influence stroke prognosis (Pilato et al., 2020). In our study, we included eligible patients who were admitted to either the Department of Neurosurgery or the Department of Neurology in West China Hospital; both departments are high-volume stroke centers, as well as diagnosis and treatment centers for complicated and severe neurological diseases in Southwest China. All the included patients were treated according to current guidelines.

However, this present study has several limitations. There might have been selection bias because not all patients had an MRI; thus, the findings could only be generalized to patients with ICH who undergo MRI in clinical practice. Since the included sample size was relatively small, we were unable to test the influence of individual CMB markers and the cumulative CSVD score on this long-term prognosis in each of the ICH subtypes. Finally, we followed after discharge, and stroke recurrence was confirmed by asking patients or their family members, so the possibility of recall bias could not be excluded. In future, large studies are needed to verify our results.

## Conclusion

We concluded that the increasing burden of PWMH, CMB, and combined CSVD burden exerts important influence on poor functional outcome and the stroke recurrence in patients with ICH. CMBs ≥ 10 and the combined CSVD burden are significantly related to time-varying survival. Our findings are important to stratify patients with ICH who are more prone to poor clinical outcomes. Our data demonstrated that MRI is valuable in assessing prognosis in patients with primary ICH.

## Data Availability Statement

The raw data supporting the conclusions of this article will be made available by the authors, without undue reservation.

## Ethics Statement

The studies involving human participants were reviewed and approved by Ethics Committee on Biomedical Research, West China Hospital of Sichuan University. The patients/participants provided their written informed consent to participate in this study.

## Author Contributions

MX and ML designed the study. MX, BL, DZ, YC, and QW enrolled and followed subjects. MX and YC reviewed clinical head CTs and MRIs for radiologic grading. MX carried out data analysis and wrote the manuscript. Important data analysis suggestions and manuscript revisions were made by BW, ML, ShuZ, and ShiZ. All authors contributed to the article and approved the submitted version.

## Conflict of Interest

The authors declare that the research was conducted in the absence of any commercial or financial relationships that could be construed as a potential conflict of interest.

## Abbreviations

CSVD: cerebral small vessel disease
ICH: intracerebral hemorrhage
WMH: white matter hyperintensities
CMB: cerebral microbleed
HA: hypertensive arteriopathy
CAA: cerebral amyloid angiopathy
EPVS: enlarged perivascular spaces
IVH: intraventricular hemorrhage
GCS: Glasgow Coma Scale
DWMH: deep WMH
PWMH: periventricular WMH
BG: basal ganglia
CSO: centrum semiovale
SWI: susceptibility-weighted imaging
IQR: interquartile range
FLAIR: fluid-attenuated inversion recovery
adOR: adjusted OR
mRS: Modified Rankin Scale
ROC: receiver operating characteristic
AUC: area under the ROC curve.
