# Supplementary material for: Cerebral Small Vessel Disease Load Predicts Functional Outcome and Stroke Recurrence After Intracerebral Hemorrhage: A Median Follow-Up of 5 Years
Source: Front Aging Neurosci. 2021 Feb 19;13:628271. doi: 10.3389/fnagi.2021.628271 (PMC7933464; doi:10.3389/fnagi.2021.628271)
Supplement: Supplementary file 1 [file Table_1.DOCX]

Supplementary table 1: Definition of complication

| **Complication** | **Definition** |
| --- | --- |
| 1. Pneumonia | Auscultatory respiratory crackles combined with at least 1 of the following: temperature >38°C, new purulent sputum, or positive chest radiograph. |
| 2. Urinary tract infection | Clinical symptoms of urinary tract infection combined with a positive urine dipstick examination for nitrite and/or pyuria. |
| 3. Gastrointestinal  Bleeding | An episode of upper or lower gastrointestinal bleeding, regardless of the cause of bleeding. |
| 4. Seizure | Clinical diagnosis of focal and/or generalized seizure in a previously nonepileptic patient. |
| 5. Septic shock | Sepsis-induced hypotension persisting despite adequate fluid  resuscitation. |
| 6. Electrolyte disturbances | Abnormal serum potassium or sodium concentration (normokalemia was defined as serum K+ concentration 3.5–5.5 mmol/l and normonatremia was defined as serum Na+ concentration 135–150 mmol/l, respectively). |
| 7. Deep vein thrombosis | Clinical diagnosis of deep vein thrombosis supported by ultrasound or venography. |
| 8. Other infections | Clinical symptoms, signs, or both associated with specific systems and positive microbiological cultures or radiographic or other imaging investigation indicating an infection other than in the chest or urinary tract. |
| 9. Depression | Low mood considered to interfere with daily activities or require pharmacological or psychiatric intervention. |
| 10. Anxiety | Symptoms of anxiety considered to interfere with daily activities or requiring pharmacological or psychiatric intervention. |
