# Supplementary material for: Cerebral Small Vessel Disease Load Predicts Functional Outcome and Stroke Recurrence After Intracerebral Hemorrhage: A Median Follow-Up of 5 Years
Source: Front Aging Neurosci. 2021 Feb 19;13:628271. doi: 10.3389/fnagi.2021.628271 (PMC7933464; doi:10.3389/fnagi.2021.628271)
Supplement: Supplementary file 2 [file Table_2.DOCX]

Supplementary table 2: Comparison between completers and patients lost to follow-up

| **Variables** | **Completers,**  **n=153** | **Patients lost to follow-up,**  **n=15** | **p** |
| --- | --- | --- | --- |
| Age, y; mean±SD | 61.4±12.3 | 56.2±10.5 | 0.113 |
| Male, n (%) | 111 (72.5) | 13 (86.7) | 0.358* |
| **ICH subtypes, n (%)** | | | |
| HA-ICH | 61 (39.9) | 8 (53.3) | 0.075* |
| CAA-ICH | 23 (15.0) | 1 (6.7) |  |
| Mixed-location ICH | 66 (43.1) | 4 (26.7) |  |
| Undetermined | 3 (2.0) | 2 (13.3) |  |
| **ICH characteristics** | | | |
| Infratentorial ICH, n (%) | 24 (15.7) | 5 (33.3) | 0.142* |
| GCS score^†^, mean±SD | 13.3±2.6 | 14.2±1.3 | 0.029 |
| Hematoma volume^‡^, median (IQR) | 10.0 (3.3-20.0) | 6.7 (3.4-28.1) | 0.783 |
| IVH, n (%) | 36 (23.5) | 5 (33.3) | 0.528* |
| Midline shift, n (%) | 42 (27.5) | 3 (20.0) | 0.762* |
| Surgical hematoma evacuation, n (%) | 14 (9.2) | 1 (6.7) | 1.000* |
| **Vascular risk factors at baseline** | | | |
| Systolic BP | 157.6±28.3 | 145.7±19.0 | 0.114 |
| Diastolic BP | 92.7±15.8 | 91.5±10.4 | 0.761 |
| Hypertension, n (%) | 123 (80.4) | 10 (66.7) | 0.313* |
| DM, n (%) | 16 (10.5) | 5 (33.3) | 0.025* |
| Hyperlipidemia, n (%) | 8 (5.2) | 0 | 1.000* |
| Prior stroke, n (%) | 15 (9.8) | 1 (6.7) | 1.000* |
| Smoking, n (%) | 46 (30.1) | 6 (40.0) | 0.559* |
| Alcohol consumption, n (%) | 28 (18.3) | 6 (40.0) | 0.084* |
| Cardiac disease, n (%) | 10 (6.5) | 0 | 0.602* |
| **Laboratory test, mean±SD** | | | |
| Blood glucose^\| \|^ | 6.9±2.2 | 9.1±6.0 | 0.175 |
| Albumin^\| \|^ | 41.7±4.4 | 43.3±4.0 | 0.169 |
| Cholesterol^§^ | 4.4±0.9 | 4.6±0.8 | 0.537 |
| HDL^§^ | 1.5±0.5 | 1.3±0.4 | 0.216 |
| LDL^§^ | 2.6±0.7 | 2.7±0.6 | 0.420 |
| Creatinine^\| \|^ | 82.9±39.7 | 77.3±19.5 | 0.585 |
| **Complication at baseline, n (%)** | | | |
| Any complication | 32 (20.9) | 6 (40.0) | 0.109* |
| **CSVD severity at baseline, n(%) or median (IQR) when appropriate** | | | |
| Lacune≥1 | 69 (45.1) | 9 (60.0) | 0.269 |
| Lacune≥2 | 37 (24.2) | 5 (33.3) | 0.532* |
| Lacune number | 0 (0-1) | 1 (0-2) | 0.420 |
| The presence of WMH | 73 (47.7) | 8 (53.3) | 0.678 |
| PWMH score | 1 (1-3) | 1 (1-3) | 0.852 |
| DWMH score | 1 (1-2) | 1 (1-2) | 0.786 |
| Total WMH score | 3 (2-5) | 3 (2-5) | 0.721 |
| The presence of CMB | 113 (73.9) | 10 (66.7) | 0.550* |
| CMBs ≥5 | 59 (38.6) | 6 (40.0) | 0.913 |
| CMBs ≥10 | 36 (23.5) | 3 (20.0) | 1.000* |
| Lober CMB number | 0 (0-2) | 0 (0-1) | 0.335 |
| BG EPVS >10 | 77 (50.3) | 10 (66.7) | 0.227 |
| BG EPVS >20 | 36 (23.5) | 4 (26.7) | 0.757* |
| CSO EPVS >20 | 48 (31.4) | 6 (40.0) | 0.565* |
| Cumulative CSVD score | 2 (1-3) | 3 (1-4) | 0.358 |

**Superscript**

*: Fisher’s exact test.

†: 152 patients had data of GCS score among completers.

‡: 119 patients had data of hematoma volume among completers, and 9 among those lost to follow-up.

§: 150 patients had data of cholesterol, HDL and LDL among completers.

| |: 152 patients had data of blood glucose, albumin, and creatinine among completers.
