# Supplementary material for: Cerebral Small Vessel Disease Load Predicts Functional Outcome and Stroke Recurrence After Intracerebral Hemorrhage: A Median Follow-Up of 5 Years
Source: Front Aging Neurosci. 2021 Feb 19;13:628271. doi: 10.3389/fnagi.2021.628271 (PMC7933464; doi:10.3389/fnagi.2021.628271)
Supplement: Supplementary file 3 [file Table_3.docx]

Supplementary table 3: Characteristics of the overall population in poor functional outcome analyses and stroke recurrence analyses

| **Variables** | **Poor functional outcome** (n=153) | **Stroke recuurence (n=131)** |
| --- | --- | --- |
| Age, y; mean±SD | 61.4±12.3 | 61.2±12.4 |
| Male, n (%) | 111 (72.5) | 98 (74.8) |
| **ICH subtypes, n (%)** | | |
| HA-ICH | 61 (39.9) | 55 (42) |
| CAA-ICH | 23 (15.0) | 18 (13.7) |
| Mixed-location ICH | 66 (43.1) | 55 (42) |
| Undetermined | 3 (2.0) | 3 (2.3) |
| **ICH characteristics** | | |
| Infratentorial ICH, n (%) | 24 (15.7) | 21 (16) |
| GCS score^*^, mean±SD | 13.3±2.6 | 13.3±2.7 |
| Hematoma volume^†^, median (IQR) | 10.0 (3.3-20.0) | 10.0 (4.2-19.8) |
| IVH, n (%) | 36 (23.5) | 33 (25.2) |
| Midline shift, n (%) | 42 (27.5) | 37 (28.2) |
| Surgical hematoma evacuation, n (%) | 14 (9.2) | 12 (9.2) |
| **Vascular risk factors at baseline** | | |
| Systolic BP, mean±SD | 157.6±28.3 | 159.5±28.8 |
| Diastolic BP, mean±SD | 92.7±15.8 | 93.3±16.3 |
| Hypertension, n (%) | 123 (80.4) | 108 (82.4) |
| Diabetes mellitus, n (%) | 16 (10.5) | 14 (10.7) |
| Hyperlipidemia, n (%) | 8 (5.2) | 6 (4.6) |
| Prior stroke, n (%) | 15 (9.8) | NA |
| Smoking, n (%) | 46 (30.1) | 41 (31.3) |
| Alcohol consumption, n (%) | 28 (18.3) | 24 (18.3) |
| Cardiac disease, n (%) | 10 (6.5) | 8 (6.1) |
| **Laboratory test, mean±SD** | | |
| Blood glucose^§^ | 6.9±2.2 | 7.0±2.3 |
| Albumin^§^ | 41.7±4.4 | 42.0±4.3 |
| Cholesterol^‡^ | 4.4±0.9 | 4.5±0.9 |
| HDL^‡^ | 1.5±0.5 | 1.5±0.5 |
| LDL^‡^ | 2.6±0.7 | 2.6±0.7 |
| Creatinine^§^ | 82.9±39.7 | 81.2±32.5 |
| **Complication at baseline, n(%)** | | |
| Any complication | 32 (20.9) | 24 (18.3) |
| **CSVD severity at baseline, n(%) or median (IQR) when appropriate** | | |
| Lacune≥1 | 69 (45.1) | 57 (43.5) |
| Lacune≥2 | 37 (24.2) | 31 (23.7) |
| Lacune number | 0 (0-1) | 0 (0-1) |
| The presence of WMH | 73 (47.7) | 60 (45.8) |
| PWMH score | 1 (1-3) | 1 (1-3) |
| DWMH score | 2 (1-2) | 1 (1-2) |
| Total WMH score | 3 (2-5) | 3 (2-4) |
| The presence of CMB | 113 (73.9) | 95 (72.5) |
| CMBs ≥5 | 59 (38.6) | 48 (36.6) |
| CMBs ≥10 | 36 (23.5) | 30 (22.9) |
| Lober CMB number | 0 (0-2) | 0 (0-2) |
| BG EPVS >10 | 77 (50.3) | 66 (50.4) |
| BG EPVS >20 | 36 (23.5) | 29 (22.1) |
| CSO EPVS >20 | 48 (31.4) | 38 (29.0) |
| Cumulative CSVD score | 2 (1-3) | 2 (1-3) |

**Abbreviations**

ICH = intracerebral hemorrhage; HA = hypertensive arteriopathy; CAA = cerebral amyloid angiopathy; GCS = Glasgow Coma Scale; BP = blood pressure; SD= standard deviation; IQR = interquartile range; IVH = intraventricular hemorrhage; HDL= high-density lipoprotein; LDL= low-density lipoprotein; CSVD = cerebral small vessel disease; WMH = white matter hyperintensities; PWMH = periventricular WMH; DWMH = deep WMH; EPVS = enlarged perivascular spaces; BG = basal ganglia; CSO = centrum semiovale; CMB = cerebral microbleed.

**Superscript**

* : 152 patients had data of GCS score in the analysis of poor functional outcome, and 130 in the analysis of stroke recurrence.

†: 119 patients had data of hematoma volume in the analysis of poor functional outcome, and 106 in the analysis of stroke recurrence.

‡: 150 patients had data of cholesterol, HDL and LDL in the analysis of poor functional outcome, and 129 patients had those data in the analysis of stroke recurrence.

§: 152 patients had data of blood glucose, albumin, and creatinine in the analysis of poor functional outcome.
