# Supplementary material for: Cerebral Small Vessel Disease Load Predicts Functional Outcome and Stroke Recurrence After Intracerebral Hemorrhage: A Median Follow-Up of 5 Years
Source: Front Aging Neurosci. 2021 Feb 19;13:628271. doi: 10.3389/fnagi.2021.628271 (PMC7933464; doi:10.3389/fnagi.2021.628271)
Supplement: Supplementary file 4 [file Table_4.DOCX]

Supplementary table 4: CSVD burden for predicting poor functional outcome and stroke recurrence.

|  | **AUC (95% CI), p value** | **p value for the differences between two AUCs*** |
| --- | --- | --- |
| **Poor functional outcome** | | |
| PWMH score | 0.687 (0.597-0.777), <0.001 | **ref** |
| Total WMH score | 0.688 (0.597-0.778), <0.001 | 0.975 (vs PWMH score) |
| CMB≥5 | 0.582 (0.482-0.682), 0.109 | 0.056 (vs PWMH score) |
| CMB≥10 | 0.580 (0.479-0.682), 0.115 | 0.051 (vs PWMH score) |
| Cumulative CSVD score | 0.652 (0.561-0.743), 0.003 | 0.414 (vs PWMH score),  0.141 (vs CMB≥10),  0.142 (vs CMB≥5) |
| CMB≥10+ PWMH | 0.704 (0.616-0.792), <0.001 | 0.347 (vs PWMH score),  **0.003** (vs CMB≥10),  **0.007** (vs CMB≥5) |
| **Stroke recurrence** | | |
| PWMH score | 0.735 (0.600-0.870), 0.004 | **ref** |
| Total WMH score | 0.704 (0.535-0.872), 0.013 | 0.314 (vs PWMH score) |
| Lobar CMB severity | 0.679 (0.522-0.837), 0.029 | 0.315 (vs PWMH score) |
| Cumulative CSVD score | 0.721 (0.580-0.862), 0.007 | 0.834 (vs PWMH score),  0.429 (vs lobar CMB severity) |
| PWMH + lobar CMB severity | 0.734 (0.580-0.887), 0.004 | 0.957 (vs PWMH score),  0.124 (vs lobar CMB severity) |

*Delong et al. test
